# Isolation of *Francisella tularensis* from Skin Ulcer after a Tick Bite, Austria, 2020

**DOI:** 10.3390/microorganisms9071407

**Published:** 2021-06-29

**Authors:** Mateusz Markowicz, Anna-Margarita Schötta, Freya Penatzer, Christoph Matscheko, Gerold Stanek, Hannes Stockinger, Josef Riedler

**Affiliations:** 1 Center for Pathophysiology, Infectiology and Immunology, Institute for Hygiene and Applied Immunology, Medical University of Vienna, Kinderspitalgasse 15, A-1090 Vienna, Austria; anna-margarita.schoetta@meduniwien.ac.at (A.-M.S.); gerold.stanek@meduniwien.ac.at (G.S.); hannes.stockinger@meduniwien.ac.at (H.S.); 2 Kardinal Schwarzenberg Klinikum, Kardinal Schwarzenbergplatz 1, A-5620 Schwarzach, Austria; freya.penatzer@gmx.at (F.P.); christoph.matscheko@ks-klinikum.at (C.M.); josef.riedler@ks-klinikum.at (J.R.)

**Keywords:** tularemia, *Francisella tularensis*, tick, multi locus sequence typing

## Abstract

Ulceroglandular tularemia is caused by the transmission of *Francisella tularensis* by arthropods to a human host. We report a case of tick-borne tularemia in Austria which was followed by an abscess formation in a lymph node, making drainage necessary. *F. tularensis* subsp. *holarctica* was identified by PCR and multilocus sequence typing.

Depending on the transmission route of *Francisella tularensis*, tularemia can present as a local infection or a systemic disease [1]. Transmission of the pathogen takes place by contact with infected animals, by bites of arthropods or through contaminated water and soil. Hares and wild rabbits are the main reservoirs of the pathogen in Austria [2]. According to the Austrian Agency for Food and Health Safety, an accumulation of fatal cases in hares was observed in the district of Salzburg in 2018 [3]. We report a case of ulceroglandular tularemia after a tick bite which occurred in Austria in the same district. Knowledge about local epidemiology of tularemia is crucial for correct diagnosis and treatment, given the wide range of various pathogens which can be transmitted by ticks to humans.

## 1. The Patient

**Figure 1:**
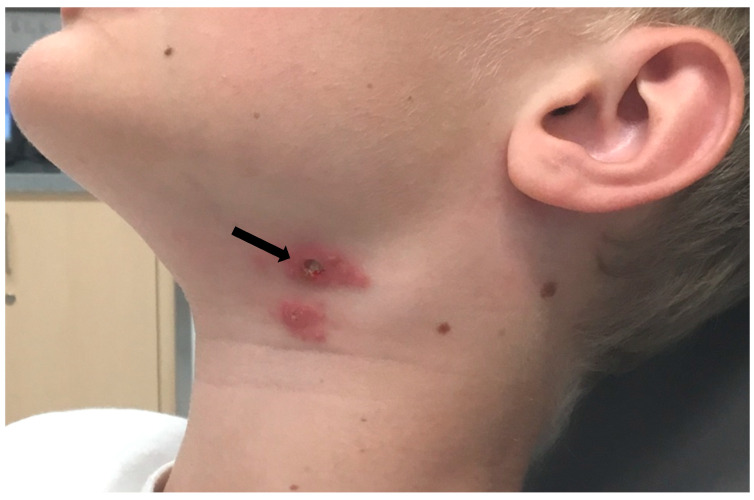
Clinical presentation of ulceroglandular tularemia after tick bite. The ulceration on the site of the tick bite is indicated by an arrow. Lymphadenopathy visible right from the inoculation site.

At the end of June 2020, a 10-year-old boy from the district of Salzburg experienced a tick bite on his neck. The boy was playing near a rabbit hatch, but he had no contact with the animals. On the same day when the tick was removed, he was subfebrile, and the site of the tick bite appeared swollen and red. The skin lesion suggested a local infection, and treatment with amoxicillin for 10 days was started. However, a few days later, ulceration on the site of the tick bite and submandibular lymphadenopathy developed (Figure 1).

Physical examination revealed small, circumscribed, raised redness with a central crust on day 8 after the tick bite. Purulent discharge was sent for routine microbiological testing and methicillin-resistant *Staphylococcus epidermidis* was cultured. Sonography showed an enlarged left lymph node at the caudal parotid pole with no signs of abscess and several reactive lymph nodes in the left vascular nerve sheath. The treatment was changed to topical fusidic acid and alcohol-based wound cleaning. Two and a half weeks after the tick bite, the patient developed abscessing lymphadenopathy, and the antibiotic treatment was changed to ampicillin/sulbactam. Abscess drainage became necessary, but no growth of pathogens was observed in a conventional culture. At this time point, the laboratory diagnostic tests revealed a moderately elevated leukocyte count (11.2/nL), a normal red cell and platelet count, a normal C-reactive protein and unremarkable liver function parameters. Serologic testing for tick-borne infections was carried out and elevated antibodies to *F. tularensis* were found by an agglutination assay (CCPro, Oberdorla, Germany) and ELISA (Virion/Serion, Würzburg, Germany) (Table 1). 

*Rickettsia* spp. infection was ruled out by an immunofluorescence assay (Focus Diagnostics, Cypress, CA, USA), and a PCR from skin crust obtained from the site of the tick bite yielded a negative result. As infection with *F. tularensis* was suspected, whole blood and the skin crust were subjected to PCR testing. A real-time PCR kit (NZYTech, Lisboa, Portugal) targeting the succinate dehygdrogenase (sdhA) gene was used. The DNA isolate from the skin specimen yielded a positive result, while the blood was negative. To further characterize the subspecies, multilocus sequence typing (MLST) was attempted as previously described [4]. This MLST scheme employs seven genes. Only three out of these seven genes (tpiA, trpE and uup) yielded a specific result after sequencing and were submitted to GenBank (Acc. No. MZ031935-MZ031937). When comparing the obtained sequences to the NCBI database by BLAST search (https://blast.ncbi.nlm.nih.gov/Blast.cgi, accessed on 1 March 2021), we identified the skin sample as containing *F. tularensis* subsp. *holarctica.* The final treatment with ciprofloxacin 500 mg b.i.d. for 10 days was highly effective, and a rapid resolution of symptoms was achieved. Written consent to publish the report was obtained from the mother of the patient.

## 2. Conclusions

In Europe, *F. tularensis* subsp. *holarctica* is the only agent of tularemia among the four other known subspecies of the zoonotic bacterium recognized worldwide [5]. Given the low seroprevalence to *F. tularensis* of 0.5% in healthy individuals [6] and sporadic cases reported each year [7,8], the infections may be considered rare in Austria. Based on the registry of the Federal Ministry of Labour, Social Affairs, Health and Consumer Protection [7], the mean number of cases per year reported from 2015 to 2019 was 11 (range 4–20 cases per year). Most cases were reported in Lower Austria, Upper Austria and Tyrol. With regard to transmission by ticks, such infections occur rarely in relation to other tick-borne infections, which we recently demonstrated in a study assessing the risk for tick-borne diseases after a tick bite [9]. Among 482 participants bitten by 1295 ticks, no patients with symptoms suggestive of tularemia were observed. A small case series of tick-borne tularemia was recently reported in Western Austria [10]. The diagnosis was made by serological testing and by molecular identification of the pathogen in a lymph node. From a practical point of view, the invasive character of such a procedure is a critical limitation to PCR testing in the outpatient area and in children. Application of skin swabs from the ulcer to identify *F. tularensis* by PCR was reported during an outbreak of ulceroglandular tularemia in Sweden [11]. Obviously, a skin crust from the ulcer is also an adequate specimen for this purpose, which we demonstrate with our case. Culture of *F. tularensis* is a possible alternative for a direct identification of the pathogen, but it can be only carried out in a BSL-3 laboratory because of the high virulence of the pathogen. Moreover, the sensitivity of PCR was shown to be superior compared to the culture [12]. 

Serologic testing was helpful to make the diagnosis of the infection in our patient (Table 1). The agglutination assay and the IgG and IgM ELISA were positive nearly three weeks after the infection. The highest levels of antibodies were observed two months after the infection, and they were still positive 10 months thereafter (Table 1). These findings are in line with a recent validation study showing a 100% sensitivity of the ELISA used in our laboratory three weeks after onset of symptoms [13]. We also found elevated antibodies to *Bartonella henselae* in an indirect immunofluorescence assay, likely reflecting an old infection, and an increase of IgM to *Borrelia burgdorferi* sensu lato in a follow-up sample, although without symptoms typical for Lyme borreliosis. 

Due to a lack of DNA extract, we were not able to repeat the investigation of missing genes, and therefore we could not provide a full MLST analysis of our isolate that would have allowed us to draw further phylogeographical and phylogenetic conclusions. However, the three allele sequences of the MLST scheme obtained during this investigation are useful for future analysis, and we therefore made them publicly available through GenBank in the NCBI database. The analysis to the species level is consistent with the reported epidemiology of *F. tularensis*, as *F. tularensis* subsp. *holartica* is the only species responsible for human infection in Europe. Thus far, molecular data on *F. tularensis* isolates in Austria are scarce. An analysis of 9 *F. tularensis* subsp. *holartica* isolates [14] by Gyuranecz et al. assigned these isolates to group B.13, which is prevalent in central and eastern European countries [15].

*F. tularensis* is an emerging pathogen in Europe, and prevention of infections is difficult because of the wide range of infection sources and routes of transmission. In the case of ulceroglandular manifestations, rapid diagnosis is recommended by molecular testing of skin specimens, which allows obviating invasive procedures such as biopsies or the surgical removal of lymph nodes.

## Figures and Tables

**Table 1:** Serologic test results of the patient with ulceroglandular tularaemia after the tick bite at the end of June 2020.

	21 July 2020	5 August 2020	10 September 2020	8 April 2021
*F. tularensis* (agglutination assay, positive ≥1:80)	1:160	1:320	1:320	1:160
*F. tularensis* IgG (EIA, positive ≥1:80)	117	194	>300 U/ml	213
*F. tularensis* IgM (EIA, positive ≥1:80)	176	214	>400 U/ml	171
*R. conorii* IgG (IIFT, positive ≥1:64)	<1:64	<1:64	np.	np.
*R. conorii* IgM (IIFT, positive ≥1:64)	<1:64	<1:64	np.	np.
*B. henselae* IgG (IIFT, positive ≥1:320)	1:1000	1:1000	np.	np.
*Borrelia burgdorferi* sl IgG (EIA, positive ≥22 RE/mL)	4	4	np.	np.
*Borrelia burgdorferi* sl IgM (EIA, positive ≥22 RE/mL)	15	39	np.	np.

np.: not performed.

## Data Availability

Not applicable.
